# Altered oscillatory brain dynamics after repeated traumatic stress

**DOI:** 10.1186/1471-244X-7-56

**Published:** 2007-10-17

**Authors:** Iris-Tatjana Kolassa, Christian Wienbruch, Frank Neuner, Maggie Schauer, Martina Ruf, Michael Odenwald, Thomas Elbert

**Affiliations:** 1 Department of Psychology, Clinical & Neuropsychology, University of Konstanz, 78457 Konstanz, Germany

## Abstract

**Background:**

Repeated traumatic experiences, e.g. torture and war, lead to functional and structural cerebral changes, which should be detectable in cortical dynamics. Abnormal slow waves produced within circumscribed brain regions during a resting state have been associated with lesioned neural circuitry in neurological disorders and more recently also in mental illness.

**Methods:**

Using magnetoencephalographic (MEG-based) source imaging, we mapped abnormal distributions of generators of slow waves in 97 survivors of torture and war with posttraumatic stress disorder (PTSD) in comparison to 97 controls.

**Results:**

PTSD patients showed elevated production of focally generated slow waves (1–4 Hz), particularly in left temporal brain regions, with peak activities in the region of the insula. Furthermore, differential slow wave activity in right frontal areas was found in PTSD patients compared to controls.

**Conclusion:**

The insula, as a site of multimodal convergence, could play a key role in understanding the pathophysiology of PTSD, possibly accounting for what has been called posttraumatic alexithymia, i.e., reduced ability to identify, express and regulate emotional responses to reminders of traumatic events. Differences in activity in right frontal areas may indicate a dysfunctional PFC, which may lead to diminished extinction of conditioned fear and reduced inhibition of the amygdala.

## Background

Severe traumatic experiences such as torture lead to a high likelihood of the consequent development of psychopathology in the trauma spectrum. Prevalence rates of posttraumatic stress disorder (PTSD) of 45% to over 90% have been reported in survivors of torture [1-3]. If left untreated, symptoms may persist for decades [3].

Traumatic experiences induce significant changes in brain structure and function (for an overview see [4-6]): the major brain areas involved in the pathology of PTSD are the medial prefrontal cortex (mPFC), which includes the anterior cingulate cortex (ACC), the hippocampus, and the amygdala.

With respect to structural changes, there have been reports of decreased hippocampal and ACC volumes in subjects with PTSD (for a review see [7]). Furthermore, several studies using proton magnetic resonance spectroscopy (^1^H-MRS) revealed abnormalities in hippocampal biochemistry in PTSD, commonly showing lower levels of N-acetylaspartate (NAA), an excitatory neurotransmitter associated with neuronal integrity [8], in the hippocampus and ACC of individuals with PTSD (e.g., [9,10]).

Functional neuroimaging studies in PTSD have mostly assessed brain activation in response to exposure to trauma reminders (trauma-related slides, sounds, personalized trauma scripts) or neural correlates of cognitive task-performance in PTSD (for an overview see [4-6]). These studies found failure of hippocampal function during memory tasks (e.g., [11,12]), enhanced amygdala sensitivity during exposure to trauma-related stimuli (for an overview see [13]) but also in response to fearful faces (masked [14,15] as well as overtly presented [16]). Shin et al. [17] observed a correlation between increased amygdala function and decreased mPFC function in response to traumatic reminders, indicating that a dysfunctional mPFC might fail to inhibit an overresponsive amygdala in PTSD. Furthermore, Rauch et al. [18] observed increased rCBF in the right paralimbic area including the insula during script-driven imagery. The insula might play an important role in anxiety disorders [19,20] and has been observed to be activated in anxiety provocation paradigms.

Whereas many brain imaging studies in PTSD have been conducted using symptom provocation paradigms, only a few studies have investigated resting state brain activity in PTSD. Using PET (positron emission tomography), Bonne et al. [21], found elevated regional cerebral blood flow (rCBF) in PTSD particularly in the cerebellum. In a SPECT (single photon emission computed tomography) study, Chung et al. [22] observed increased rCBF in limbic and decreased perfusion in the superior frontal gyrus, and parietal and temporal regions.

The functional architecture of neuronal networks is reflected in the dynamics of spontaneous neural mass activity, measurable by magnetoencephalography (MEG). MEG measurement filters widespread activity with its radial net currents but is sensitive to circumscribed activity of patches in cortical sulci. An equivalent current dipole (ECD) model provides an excellent approximation to localize such focal assemblies of active pyramidal cells [23].

Abnormally high densities of focal generators of slow waves have been found to be related to brain pathology or dysfunctional neural tissue [24-28]. Brain lesions are frequently accompanied by abnormal slow waves in the deafferented regions, for instance the penumbra after stroke [29] or in circumscribed regions around a tumor [24-27]. Focal slow waves are abundant in degenerative disorders such as Alzheimer's disease [30,31] and abnormally distributed in depression and schizophrenia [32-35]. More recently, it has been reported that dissociative experiences in survivors of prolonged torture were reflected in generators of abnormal slow waves in the left fronto-temporal cortex [36], possibly reflecting the decoupling of frontal affective processors from left perisylvian language areas. These data suggest that abnormally high densities of focal slow wave generators distinguish dysfunctional brain regions even when macroscopic structural lesions or functional alterations are not readily detectable by other imaging techniques.

The present study investigates alterations in spontaneous brain activity in victims of severe and multiple extreme stressors, including torture. Magnetic source imaging was applied to map local clustering of slow wave generators in the brains of survivors of severe and repeated torture fulfilling DSM-IV criteria of posttraumatic stress disorder. Given the previous observations, we expected more slow waves in frontal and temporal brain regions in PTSD patients compared to controls.

## Methods

### Participants

194 subjects participated in the study: 97 healthy controls (mean age 30.6 years, *SD *= 10.3, age range 22–66; 56 male, 41 female) and 97 patients (mean age 31.9 years, *SD *= 7.9, age range 16–53; 64 male, 33 female) diagnosed with PTSD according to DSM-IV [37]. All persons were right-handed as measured by the Edinburgh handedness questionnaire [38].

Participants with posttraumatic stress disorder (PTSD) were refugees who came for treatment or expert opinion to the Psychotrauma Research and Outpatient Clinic for Refugees, located at the Center for Psychiatry, Reichenau, Germany. Refugees had the following ethnicities: 74 Turks (71 Kurds), 6 Albanians, 3 Algerians, 3 Romanies, 1 Amharic (Ethiopian), 1 Bosnian, 1 Cameroonian, 1 Georgian, 1 Iranian, 1 Liberian, 1 Serb, 1 Sierra Leonian, and 1 Tamil, and 2 Germans who had fled from prisons of the former German Democratic Republic. In clinical interviews with trained translators, trained psychologists completed the Posttraumatic Stress Diagnostic Scale [PDS, 39], the Hopkins Symptoms Checklist-25 [HSCL-25, 40], or the interviewer-rated 21-item version of the original Hamilton Depression Scale [HAM-D, 41]. See Table 1 for mean questionnaire scores and standard deviations. For 40 patients HAM-D scores were available as a measure of depressive symptoms and for 57 patients HSCL scores were available. Fifty-five percent of participants with PTSD had received blows in the face, 24 were severely beaten on the head and sustained injuries, so that concussions could not be excluded, and 18 were not beaten on the head. Two subjects abused alcohol, 2 subjects abused drugs, and 1 subject abused both alcohol and drugs. Major depression is highly comorbid with PTSD [42], in particular in very severe cases of PTSD such as in survivors of torture. Due to substantial symptom overlap between severe PTSD and depression (disordered sleep, anhedonia/emotional numbing, concentration problems, irritability), comorbid depression diagnosis should be treated with caution and therefore quantitative depression scores were included in the analyses. In fact, nearly all participants would have additionally met DSM-IV criteria for major depression.

**Table 1:** Questionnaire values

		PTSD Group		
		
Questionnaire	Subscale	*N*	*M*	*SD*
PDS	Total Score (PDS_Sum_)	97	35.55	7.02
	Intrusions (PDS_I_)	97	10.71	3.02
	Avoidance (PDS_A_)	97	13.83	4.04
	Hyperarousal (PDS_H_)	97	10.86	2.81
HSCL-25	Total Score (HSCL_Sum_)	57	2.97	0.47
	Anxiety (HSCL_A_)	57	2.97	0.56
	Depression (HSCL_D_)	57	2.99	0.52
HAM-D		40	23.37	7.67

Mean Questionnaire Values (*M*) and Standard Deviations (*SD*) of PTSD Patients. PDS, Posttraumatic Stress Diagnostic Scale; HSCL-25, Hopkins Symptoms Checklist-25; HAM-D, Hamilton Depression Scale.

Controls were recruited by public newspaper announcements and on campus bulletin boards. In order to participate in the study, controls had to have no current or past history of psychiatric disorders and had to be free of psychiatric medication. All participants provided written informed consent and the procedures were approved by the ethics committee of the University of Konstanz. Controls were paid 5 EUR per hour for participation.

### Assessment and analysis of MEG

The magnetoencephalogram (MEG) was measured in supine position with a 148-channel whole-head neuromagnetometer (MAGNES™ 2500 WH, 4D Neuroimaging, San Diego, CA, USA) during a 5 min resting period before other experimental investigations (not reported in this manuscript) were conducted. Subjects were instructed to relax but stay awake and fixate a mark on the ceiling of the room throughout the recording period, in order to avoid eye and head movements. A video camera monitored subjects' behavior and assured compliance throughout the experiment. In order to define a subject-related headframe coordinate system and head shapes of subjects, five index points were digitized with a Polhemus 3Space^® ^Fasttrack (Polhemus, Colchester, VT, USA) prior to each measurement. The subject's head position relative to the pickup coils of the sensor was estimated before and after each measurement.

MEG was recorded with a sampling rate of 678.17 Hz and a band-pass filter of 0.1–200 Hz. The electro-oculogram (EOG) was recorded with 2 electrodes attached to the left and right outer canthus of the right eye and 2 electrodes attached below and above the right eye. In addition, the electrocardiogram (ECG) was monitored with 2 electrodes attached to the right collarbone and the lowest left rib. ECG and EOG data were acquired using Synamps amplifiers (NEUROSCAN, Compumedics Germany GmbH, Hamburg, Germany). Data were visually inspected for eye blink and eye movement artifacts. While magnetocardiogram (MCG) artifacts were corrected by using the *cardiac remover *(part of the Whole Head System software, version 1.2.5; 4D Neuroimaging), time segments contaminated by EOG artifacts were excluded from further analysis.

### Data reduction and analysis

Data acquired during the 5 min resting period were reduced by a factor of 16 (antialias filters were applied automatically in the same processing step). Altogether this results in (678.17 Hz × 300 s)/16 = 12715 sampling points. Data were digitally filtered in the delta (1.5–4.0 Hz) frequency range with a Butterworth filter of order 2. In each one of five standard channel groups (as defined by 4D Neuroimaging: anterior, center, posterior, left, and right), a single equivalent current dipole (ECD) was fitted for each time point in the selected artifact free segments. Groups did not differ significantly in the number of artifact free sampling points. Dipoles were assigned to 1331 2 × 2 × 2 cm^3 ^voxels (for details on the method see [43]). Fitted dipoles had to satisfy the following criteria: a) goodness of fit (GoF) > 90%, b) source intensity of 10–100nAm around a focal point source (equivalent to 0.1–1 cm^2 ^of activated cortex). Further analysis comprised two different strategies: voxel-based and mask-based.

#### Voxel-based analysis

Dipole density was estimated for each subject within each voxel of the source volume by calculating the average number of dipoles per time unit in the voxel over artifact free segments and divided by the number of artifact free sampling points (coded as NARTFREE). The result is termed „voxel-based dipole density for the i-th voxel“ (VBDD_i_). To obtain a normal distribution across subjects, LVBDD_i _was calculated by taking the logarithm of VBDD_i _and setting LVBDD_i _to zero for empty voxels. For further analysis of individual subjects and visualization, LVBDD_i _was *z*-transformed using the mean and standard deviation of LVBDD_i _in the control group. The result is denoted ZLVBDD_i_.

**Figure 1:**
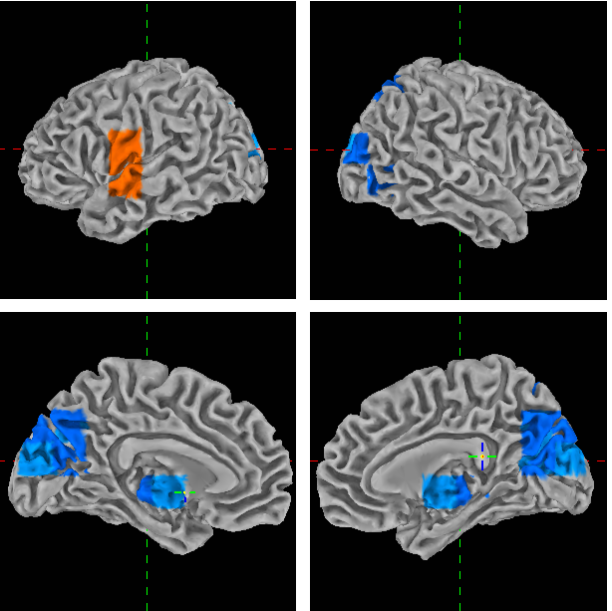
**Voxel-based analysis**. Differences of ZLVBDD_i _(z-transformed logarithm voxel-based dipole density for the i-th voxel) for the left and right hemisphere, medial (top) and lateral (bottom) view. Only voxels with a significance value of *t *> 3.42, *p *< .0008 are shown. Orange voxels indicate more focal slow waves in the PTSD than in the control group. Blue voxels indicate less focal slow waves in the PTSD than in the control group.

Subjects' MEG data were aligned by using their individual headframe coordinate system defined through the nasion point and the two ear canals. A *t*-value difference map was calculated comparing ZLVBDD_i _values of PTSD patients and controls on a voxel-by-voxel basis. Using AFNI (Analysis of functional neuroimages [44]) the *t*-value difference map was overlaid with the CH2 brain template (see Figure 1).

#### Mask-based analysis

Dipole density VBDD_i _was averaged over each one of eight regions based on anatomically defined brain masks, generated for temporal, frontal, central and parieto-occipital parts of the brain within each hemisphere following the classification of the anatomical atlas provided with MRICRO's AAL [45]. Empty voxels did not contribute to averages over regions. The result is the mask-based dipole density in region r and hemisphere h (MBDD_rh_). MBDD_rh _of empty region-hemisphere combinations was set to half the number of artifact-free epochs of a subject for easier algorithmic calculation [43]. For further statistical analysis, MBDD_rh _was logarithmized and *z*-transformed using the mask-based mean and standard deviation in the control group. The result is denoted for simplicity in the following as *z *values.

#### Max values

Maximal dipole densities within each region-hemisphere combination were calculated by setting Max_rh _to the maximal *z*-value over all voxels in region r and hemisphere h.

#### AvgAbThre values

The average dipole density above the threshold of *z *= 2 within region r and hemisphere h was defined as the average dipole density over all voxels in region r and hemisphere h where the density exceeded *z *= 2.

### Statistical analysis

For data analysis, linear mixed effects models [46] were implemented using SAS 9.1 (SAS Institute Inc.). In all analyses of variance (ANOVAs), Subjects served as a random effect nested in Gender and Group [47], whereas all other factors were fixed effects. Differences in dipole density solutions between groups were evaluated by means of a 2 × 2 × 4 × 2 ANOVA with between factors Group (PTSD patients, controls), Gender (male, female), and repeated measures factors Region (frontal, central, temporal, parieto-occipital) and Hemisphere (left, right).

Because Max and AvgAbThre values cannot be assumed to be normally distributed, ANOVA *F *statistics will not be *F *distributed for these dependent variables. In order to detect significant effects, permutation tests were conducted on the residuals of submodels for each factor and interaction [48,49], holding all other factors and interactions constant by use of restricted permutations. For example, when investigating the significance of Group × Region × Hemisphere, the vectors of eight residuals corresponding to the eight combinations of Region and Hemisphere were permuted within each subject before being added to the unpermuted values predicted by the submodel defined by excluding Group × Region × Hemisphere from the full model. Next, subjects' group designations were permuted among male and female subjects separately, in order not to include gender effects. The resulting resampled dependent variable datasets were analyzed using the full model. In each case, 1000 permutations were conducted, and the original *F *value was inserted in the empirical distribution of *F *values from the resampled ANOVAs. *p *values as reported below are the difference between 1 and this percentile, such that an original *F *value falling at the 95^th ^percentile in the resampled *F *value distribution is considered significant at the .05 level and is reported as *p *= .05. Degrees of freedom are irrelevant in permutation tests and are not reported below. Significant effects were further analyzed by calculating contrasts, applying the same permutation procedure as reported above. For significant contrasts, Cohen's *d *was calculated as a measure of effect size, using pooled variances [50,51].

## Results

### Voxel-based analysis

Enhanced abnormal slow wave activity was observed in voxels in left temporal areas in the region of the insula in individuals with PTSD compared to controls, whereas in voxels in parieto-occipital areas fewer slow waves were observed in the PTSD compared to the control group (Figure 1).

### Mask-based analyses

#### Analysis of z-values

Permutation tests revealed a significant interaction of Group × Region, *p *< .001, whereas Group × Hemisphere failed to be significant, *p *= .10. Subsequent contrasts showed that PTSD patients exhibited smaller *z*-values than the control group over parieto-occipital areas, *p *< .001, *d *= .85, whereas over frontal, central and temporal areas no significant differences between individuals with PTSD and controls were observed.

#### Analysis of mean z-values above 2 SD threshold (AvgAbThre)

**Figure 2:**
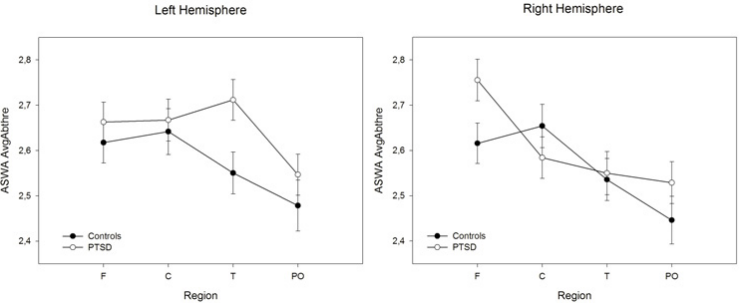
**Mask-based analysis: average above threshold**. Least Square Means of AvgAbThre (average density above a set threshold – see methods) values for each region in the left and right hemisphere in individuals with PTSD and controls.

Permutation tests showed an interaction of Group × Hemisphere × Region, *p *= .008. Contrasts revealed larger AvgAbThre values in the PTSD compared to the control group over left temporal, *p *= .003, *d *= .43, and right frontal sites, *p *= .03, *d *= .33 (see Figure 2).

#### Analysis of maximal z-values in each region (Max)

**Figure 3:**
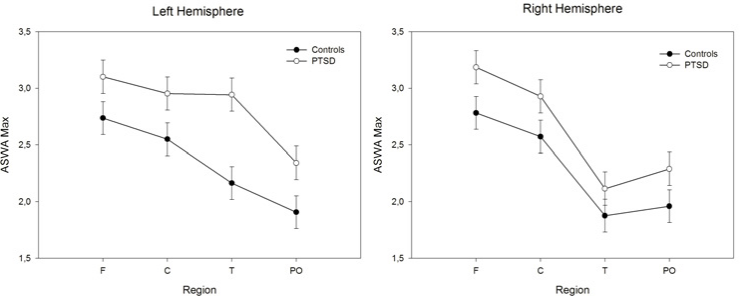
**Mask-based analysis: max values**. Least Square Means of Max values for each region in the left and right hemisphere in individuals with PTSD and controls.

Regions generating pronounced foci of slow waves, i.e. AvgAbThre values, also produced high Max values. Permutation tests revealed an interaction of Group × Hemisphere × Region, *p *= .03. Larger Max values were observed for individuals with PTSD compared to control subjects over left temporal, *p *< .001, *d *= .56, left central, *p *= .05, *d *= .30, left parietooccipital, *p *= .03, *d *= .37, and right frontal sites, *p *= .05, *d *= .30 (see Figure 3).

## Discussion

Analyzing the source of focally generated slow waves by means of magnetic source imaging revealed a highly consistent regional pattern in individuals with PTSD compared to healthy controls: PTSD patients showed higher average dipole densities above a threshold of 2 *SD *(AvgAbThre) and higher maximal dipole densities (Max) in left temporal and right frontal areas in comparison to controls. The enhanced presence of slow wave producing foci in the left intrasylvian cortex of PTSD patients compared to controls demonstrates altered functioning, possibly a dysfunction of the left insula in PTSD psychopathology. This corresponds well with the results of the voxel-wise analysis of dipole densities, which revealed significantly larger dipole densities in PTSD patients compared to controls in the left insula. Supporting such a finding, the right prefrontal cortex (PFC) together with the left insula/putamen region has been found to be specifically associated with psychological stress [52], and thus these regions might be particular targets of stress hormones, leading to structural and/or functional damage.

### Enhanced left-hemispheric slow wave activity in the intrasylvian cortex

The insula lies deep inside the lateral sulcus in the sylvian fissure under the operculum. It is an important site of multimodal convergence, directly receiving afferent auditory, visual, somatosensory, gustatory and olfactory information. The insula is involved in attention, pain perception, emotion, and in the processing of verbal, motor and musical information, as well as visceral sensory and vestibular functions. It has direct connections not only with the primary and secondary sensory areas but also with the hippocampus, the amygdala, the cingulate gyrus and Broca's area among others (for an overview see [20,53]). Functional neuroimaging studies have linked insula activation to modulation of affective processing, cognitive and affective processing during learning, and aversive interoceptive processing [19].

The role of the insula in speech and language processing has long been noted [54], while there is now broad clinical and functional imaging evidence for a participation of the left anterior insula in speech motor control [55]. The left insula is notably larger than the right in most humans [56], consistent with a left-hemispheric dominance in language. Mutism has been frequently observed in cases of insular pathology: Transient mutism is found in cases of left inferior motor cortex damage extending to the insula [57,58], whereas lasting mutism appears to be associated with bilateral lesions of the frontal operculum and anterior insula [59-61]. Functional magnetic resonance studies have revealed significant blood flow increases at the level of intrasylvian cortex during overt speech in the left anterior insula [62,63], suggesting that the left insula plays a role in the coordination of speech articulation. Because of its role in both affective and speech processing, the insula has been assumed to influence verbal affect [54]. It has been proposed [55] that the insula might fuse linguistic data structures with affective-prosodic information on a moment-to-moment basis into a smooth motor innervation pattern during speech production.

Recently, the role of the insula in anxiety disorders [19] and other neuropsychiatric disorders has received more attention [20]. Indeed, studies investigating volume changes in the brains of individuals with PTSD found evidence for reduced gray matter density in the left insula [64,65].

This insular dysfunction may account for the nonverbal nature of traumatic memory recall in PTSD subjects (compare [66]) and the difficulty to identify, verbally express, and regulate one's emotional states in response to various kinds of trauma reminders, which has been called *posttraumatic alexithymia *[67]. Frewen et al. [67] suggested that alexithymic individuals with PTSD may experience intense emotional-physiological states such as fear, anger and dysphoria that are poorly integrated with, and modulated by, higher-order verbal cognitive processing. Therefore these individuals may report that they either do not know what they feel or cannot feel anything at all. Indeed, Frewen et al. found positive correlations between the Toronto alexithymia scale (TAS-20) and PTSD symptoms, dissociation, and childhood abuse and neglect. Furthermore, in a functional magnetic resonance imaging (fMRI) study they reported positive correlations between TAS-20 scores and insula activation in individuals with PTSD when exposed to trauma script imagery. However, Frewen et al. investigated insula activity in response to a trauma script induction procedure and did not directly measure emotional identification or expression abilities and associated neural network dysfunction. Thus, it is possible that the positive correlation they find is due to the fact that more severe traumatization leads both to stronger alexithymia and more anxiety, and therefore insula activation when confronted with trauma reminders.

Furthermore, the hypoperfusion of Broca's area (motor speech) during symptom provocation in PTSD is a replicated finding [18,68]. Broca's area is necessary for the labeling of emotions, therefore, its deactivation under symptom provocation eventually may be due to insular dysfunction in connecting verbal affect with smooth motoric articulation patterns.

Alternatively, given the central role of the insula in multimodal processing, the connection between structural and functional changes in the insula and PTSD may be more complex and may touch on more facets of brain function than emotion identification and verbalization. Thus, future work should focus on elucidating the role of the insula in PTSD, along with other neuropsychiatric disorders, in which the relationship to the insula has only recently come under scrutiny [20].

### Enhanced right frontal slow wave activity

The enhanced regional slow wave clusters (AvgAbThre) in the right frontal cortex are consistent with the large body of literature on structural, neurochemical, and functional abnormalities in medial PFC, including anterior cingulate cortex (ACC) and medial frontal gyrus, in PTSD [13]. The right PFC has been associated with negative affect, behavioral inhibition and vigilant attention, whereas left-sided PFC regions are particularly involved in approach-related, appetitive goals [69].

The most prevalent functional neuroimaging finding is that of a relatively diminished responsivity in medial PFC (decreased activation and/or failure to activate) in PTSD [13], which would be consistent with the present results showing PFC dysfunction as indicated by enhanced abnormal slow waves in right frontal areas. Current models on the neurocircuitry of PTSD assume the medial PFC, which plays an important role in fear extinction, to be hyporesponsive, leading to diminished extinction of conditioned fear and together with a hyperresponsive amygdala to augmented fear responses and hyperarousal symptoms [13].

### Parieto-occipital slow wave activity

The meaning of differences in parieto-occipital delta dipole densities in PTSD subjects compared to controls in the resting state deserves further investigation. Of particular interest is the present finding of lower levels (*z *values) but higher left-hemispheric peak (Max) values and a general trend toward more abnormal slow waves over parieto-occipital areas (compare Figures 2 and 3) in PTSD patients compared to controls. This clearly points to the existence of slow wave foci that do not appear in averaged values but differentiate between severely traumatized and non-traumatized individuals. However, currently the functional significance of these findings remains unclear and should be further investigated in future studies.

## Conclusion

This study found enhanced focal slow wave activity in left temporal areas corresponding to the insular cortex. The insula as a site of multimodal convergence could play a key role in understanding the pathophysiology of PTSD, possibly accounting for what has been called posttraumatic alexithymia.

Furthermore, differential slow wave activity in right frontal areas was found in PTSD patients compared to controls. This may indicate a dysfunctional PFC, which may lead to diminished extinction of conditioned fear and reduced inhibition of the amygdala.

## Competing interests

The author(s) declare that they have no competing interests.

## Authors' contributions

ITK conducted the statistical analysis and wrote the manuscript. ITK, CW, and TE conceptualized the study and developed the ASWA methodology. ITK, FN, MS, MR, MO, and TE diagnosed and took care of PTSD patients. All authors have read and approved the final version of the manuscript.

## Pre-publication history

The pre-publication history for this paper can be accessed here:



## References

[B1] Moisander PA, Edston E (2003). Torture and its sequel – a comparison between victims from six countries. Forensic Science International.

[B2] Keller A, Lhewa D, Rosenfeld B, Sachs E, Aladjem A, Cohen I, Smith H, Porterfield K (2006). Traumatic experiences and psychological distress in an urban refugee population seeking treatment services. J Nerv Ment Dis.

[B3] Bichescu D, Schauer M, Saleptsi E, Neculau A, Elbert T, Neuner F (2005). Long-term consequences of traumatic experiences: an assessment of former political detainees in Romania. Clin Pract Epidemol Ment Health.

[B4] Bremner JD (2006). Traumatic stress: effects on the brain. Dialogues in Clinical Neuroscience.

[B5] Bremner JD (2007). Functional neuroimaging in post-traumatic stress disorder. Expert Review of Neurotherapeutics.

[B6] Kolassa I-T, Elbert T Structural and functional neuroplasticity in relation to traumatic stress. Current Directions in Psychological Science.

[B7] Karl A, Schaefer M, Malta LS, Dorfel D, Rohleder N, Werner A (2006). A meta-analysis of structural brain abnormalities in PTSD. Neurosci Biobehav Rev.

[B8] Villarreal G, Petropoulos H, Hamilton DA, Rowland LM, Horan WP, Griego JA, Moreshead M, Hart BL, Brooks WM (2002). Proton magnetic resonance spectroscopy of the hippocampus and occipital white matter in PTSD: preliminary results. Can J Psychiatry.

[B9] De Bellis MD, Keshavan MS, Spencer S, Hall J (2000). N-Acetylaspartate concentration in the anterior cingulate of maltreated children and adolescents with PTSD. American Journal of Psychiatry.

[B10] Ham BJ, Chey J, Yoon SJ, Sung Y, Jeong DU, Ju Kim S, Sim ME, Choi N, Choi IG, Renshaw PF (2007). Decreased N-acetyl-aspartate levels in anterior cingulate and hippocampus in subjects with post-traumatic stress disorder: a proton magnetic resonance spectroscopy study. The European Journal of Neuroscience.

[B11] Bremner JD, Vythilingam M, Vermetten E, Southwick SM, McGlashan T, Nazeer A, Khan S, Vaccarino LV, Soufer R, Garg PK (2003). MRI and PET study of deficits in hippocampal structure and function in women with childhood sexual abuse and posttraumatic stress disorder. American Journal of Psychiatry.

[B12] Shin LM, Shin PS, Heckers S, Krangel TS, Macklin ML, Orr SP, Lasko N, Segal E, Makris N, Richert K (2004). Hippocampal function in posttraumatic stress disorder. Hippocampus.

[B13] Shin LM, Rauch SL, Pitman RK (2006). Amygdala, medial prefrontal cortex, and hippocampal function in PTSD. Annals of the New York Academy of Sciences.

[B14] Armony JL, Corbo V, Clement MH, Brunet A (2005). Amygdala response in patients with acute PTSD to masked and unmasked emotional facial expressions. American Journal of Psychiatry.

[B15] Rauch SL, Whalen PJ, Shin LM, McInerney SC, Macklin ML, Lasko NB, Orr SP, Pitman RK (2000). Exaggerated amygdala response to masked facial stimuli in posttraumatic stress disorder: a functional MRI study. Biological Psychiatry.

[B16] Shin LM, Wright CI, Cannistraro PA, Wedig MM, McMullin K, Martis B, Macklin ML, Lasko NB, Cavanagh SR, Krangel TS (2005). A functional magnetic resonance imaging study of amygdala and medial prefrontal cortex responses to overtly presented fearful faces in posttraumatic stress disorder. Archives of General Psychiatry.

[B17] Shin LM, Orr SP, Carson MA, Rauch SL, Macklin ML, Lasko NB, Peters PM, Metzger LJ, Dougherty DD, Cannistraro PA (2004). Regional cerebral blood flow in the amygdala and medial prefrontal cortex during traumatic imagery in male and female Vietnam veterans with PTSD. Archives of General Psychiatry.

[B18] Rauch SL, van der Kolk BA, Fisler RE, Alpert NM, Orr SP, Savage CR, Fischman AJ, Jenike MA, Pitman RK (1996). A symptom provocation study of posttraumatic stress disorder using positron emission tomography and script-driven imagery. Archives of General Psychiatry.

[B19] Paulus MP, Stein MB (2006). An insular view of anxiety. Biological Psychiatry.

[B20] Nagai M, Kishi K, Kato S (2007). Insular cortex and neuropsychiatric disorders: A review of recent literature. European Psychiatry.

[B21] Bonne O, Gilboa A, Louzoun Y, Brandes D, Yona I, Lester H, Barkai G, Freedman N, Chisin R, Shalev AY (2003). Resting regional cerebral perfusion in recent posttraumatic stress disorder. Biological Psychiatry.

[B22] Chung YA, Kim SH, Chung SK, Chae JH, Yang DW, Sohn HS, Jeong J (2006). Alterations in cerebral perfusion in posttraumatic stress disorder patients without re-exposure to accident-related stimuli. Clinical Neurophysiology.

[B23] Elbert T, Andrä W, Nowak H (1998). Neuromagnetism. Magnetism in medicine.

[B24] Baayen JC, de Jongh A, Stam CJ, de Munck JC, Jonkman JJ, Trenite DG, Berendse HW, van Walsum AM, Heimans JJ, Puligheddu M (2003). Localization of slow wave activity in patients with tumor-associated epilepsy. Brain Topography.

[B25] de Jongh A, Baayen JC, de Munck JC, Heethaar RM, Vandertop WP, Stam CJ (2003). The influence of brain tumor treatment on pathological delta activity in MEG. Neuroimage.

[B26] de Jongh A, de Munck JC, Baayen JC, Jonkman EJ, Heethaar RM, van Dijk BW (2001). The localization of spontaneous brain activity: first results in patients with cerebral tumors. Clinical Neurophysiology.

[B27] Vieth JB, Kober H, Grummich P (1996). Sources of spontaneous slow waves associated with brain lesions, localized by using the MEG. Brain Topography.

[B28] Tanaka A, Kimura M, Yoshinaga S, Tomonaga M, Mizoguchi T (1998). Quantitative electroencephalographic correlates of cerebral blood flow in patients with chronic subdural hematomas. Surgical Neurology.

[B29] Meinzer M, Elbert T, Wienbruch C, Djundja D, Barthel G, Rockstroh B (2004). Intensive language training enhances brain plasticity in chronic aphasia. BMC Biology.

[B30] Fernandez A, Arrazola J, Maestu F, Amo C, Gil-Gregorio P, Wienbruch C, Ortiz T (2003). Correlations of hippocampal atrophy and focal low-frequency magnetic activity in Alzheimer disease: volumetric MR imaging-magnetoencephalographic study. AJNR American Journal of Neuroradiology.

[B31] Fernandez A, Maestu F, Amo C, Gil P, Fehr T, Wienbruch C, Rockstroh B, Elbert T, Ortiz T (2002). Focal temporoparietal slow activity in Alzheimer's disease revealed by magnetoencephalography. Biological Psychiatry.

[B32] Fehr T, Kissler J, Wienbruch C, Moratti S, Elbert T, Watzl H, Rockstroh B (2003). Source distribution of neuromagnetic slow-wave activity in schizophrenic patients – effects of activation. Schizophrenia Research.

[B33] Wienbruch C, Moratti S, Elbert T, Vogel U, Fehr T, Kissler J, Schiller A, Rockstroh B (2003). Source distribution of neuromagnetic slow wave activity in schizophrenic and depressive patients. Clinical Neurophysiology.

[B34] Fehr T, Kissler J, Moratti S, Wienbruch C, Rockstroh B, Elbert T (2001). Source distribution of neuromagnetic slow waves and MEG-delta activity in schizophrenic patients. Biological Psychiatry.

[B35] Fernandez A, Rodriguez-Palancas A, Lopez-Ibor M, Zuluaga P, Turrero A, Maestu F, Amo C, Lopez-Ibor JJ, Ortiz T (2005). Increased occipital delta dipole density in major depressive disorder determined by magnetoencephalography. Journal of Psychiatry & Neuroscience: JPN.

[B36] Ray W, Odenwald M, Neuner F, Schauer M, Ruf M, Wienbruch C, Rockstroh B, Elbert T (2006). Decoupling neural networks from reality: dissociative experiences in torture victims are reflected in abnormal brain waves in left frontal cortex. Psychological Science.

[B37] American Psychiatric Association (1994). Diagnostic and statistical manual of mental disorders.

[B38] Oldfield RC (1971). The assessment and analysis of handedness: The Edinburgh Inventory. Neuropsychologia.

[B39] Foa EB, Cashman L, Jaycox L, Perry K (1997). The validation of a self-report measure of posttraumatic stress disorder: The Posttraumatic Diagnostic Scale. Psychological Assessment.

[B40] Derogatis LR, Lipman RS, Rickels K, Uhlenhuth EH, Covi L (1974). The Hopkins Symptom Checklist: A self-report symptom inventory. Behavioral Science.

[B41] Hamilton M (1960). A rating scale for depression. Journal of Neurology, Neurosurgery and Psychiatry.

[B42] Kessler RC, Sonnega A, Bromet E, Hughes M, Nelson CB (1995). Posttraumatic stress disorder in the National Comorbidity Survey. Archives of General Psychiatry.

[B43] Wienbruch C (2007). Abnormal slow wave mapping (ASWAM) – A tool for the investigation of abnormal slow wave activity in the human brain. Journal of Neuroscience Methods.

[B44] Analysis of functional neuroimages (AFNI). http://afni.nimh.nih.gov/afni/about.

[B45] Tzourio-Mazoyer N, Landeau B, Papathanassiou D, Crivello F, Etard O, Delcroix N, Mazoyer B, Joliot M (2002). Automated anatomical labeling of activations in SPM using a macroscopic anatomical parcellation of the MNI MRI single subject brain. Neuroimage.

[B46] Laird NM, Ware JH (1982). Random-Effects Models for Longitudinal Data. Biometrics.

[B47] Winer BJ, Brown DR, Michels KM (1991). Statistical principles in experimental design.

[B48] Freedman D, Lane D (1983). A nonstochastic interpretation of reported significance levels. Journal of Business and Economic Statistics.

[B49] Anderson MJ, Legendre P (1999). An empirical comparison of permutation methods for tests of partial regression coefficients in a linear model. Journal of Statistical Computation and Simulation.

[B50] Cohen J (1988). Statistical power analysis for the behavioral sciences.

[B51] Rosnow RL, Rosenthal R (1996). Computing contrasts, effect sizes, and counternulls on other people's published data: General procedures for research consumers. Psychological Methods.

[B52] Wang J, Rao H, Wetmore GS, Furlan PM, Korczykowski M, Dinges DF, Detre JA (2005). Perfusion functional MRI reveals cerebral blood flow pattern under psychological stress. Proceedings of the National Academy of Sciences of the United States of America.

[B53] Augustine JR (1996). Circuitry and functional aspects of the insular lobe in primates including humans. Brain research Brain research reviews.

[B54] Ardila A (1999). The role of insula in language: an unsettled question. Aphasiology.

[B55] Ackermann H, Riecker A (2004). The contribution of the insula to motor aspects of speech production: a review and a hypothesis. Brain and Language.

[B56] Mesulam MM, Mufson EJ, Peters A, Jones EG (1985). The insula of Reil in man and monkey. Architectonics, connectivity and function. Cerebral Cortex.

[B57] Schiff HB, Alexander MP, Naeser MA, Galaburda AM (1983). Aphemia. Clinical-anatomic correlations. Archives of Neurology.

[B58] Alexander MP, Benson DF, Stuss DT (1989). Frontal lobes and language. Brain and Language.

[B59] Sussman NM, Gur RC, Gur RE, O'Connor MJ (1983). Mutism as a consequence of callosotomy. Journal of Neurosurgery.

[B60] Cappa SF, Guidotti M, Papagno C, Vignolo LA (1987). Speechlessness with occasional vocalization after bilateral opercular lesions: a case study. Aphasiology.

[B61] Pineda D, Ardila A (1992). Lasting mutism associated with buccofacial apraxia. Aphasiology.

[B62] Wildgruber D, Ackermann H, Klose U, Kardatzki B, Grodd W (1996). Functional lateralization of speech production at primary motor cortex: a fMRI study. Neuroreport.

[B63] Riecker A, Ackermann H, Wildgruber D, Dogil G, Grodd W (2000). Opposite hemispheric lateralization effects during speaking and singing at motor cortex, insula and cerebellum. Neuroreport.

[B64] Corbo V, Clement MH, Armony JL, Pruessner JC, Brunet A (2005). Size versus shape differences: contrasting voxel-based and volumetric analyses of the anterior cingulate cortex in individuals with acute posttraumatic stress disorder. Biological Psychiatry.

[B65] Chen S, Xia W, Li L, Liu J, He Z, Zhang Z, Yan L, Zhang J, Hu D (2006). Gray matter density reduction in the insula in fire survivors with posttraumatic stress disorder: a voxel-based morphometric study. Psychiatry Research.

[B66] Lanius RA, Williamson PC, Densmore M, Boksman K, Neufeld RW, Gati JS, Menon RS (2004). The nature of traumatic memories: a 4-T FMRI functional connectivity analysis. American Journal of Psychiatry.

[B67] Frewen PA, Pain C, Dozois DJ, Lanius RA (2006). Alexithymia in PTSD: psychometric and FMRI studies. Annals of the New York Academy of Sciences.

[B68] Shin LM, McNally RJ, Kosslyn SM, Thompson WL, Rauch SL, Alpert NM, Metzger LJ, Lasko NB, Orr SP, Pitman RK (1997). A positron emission tomographic study of symptom provocation in PTSD. Annals of the New York Academy of Sciences.

[B69] Davidson RJ (2003). Darwin and the neural bases of emotion and affective style. Annals of the New York Academy of Sciences.

